# “We All Join Hands”: Perceptions of the Kangaroo Method Among Female Relatives of Newborns in The Gambia

**DOI:** 10.1177/1049732320976365

**Published:** 2020-12-08

**Authors:** Helen Brotherton, Maura Daly, Penda Johm, Bintou Jarju, Joanna Schellenberg, Loveday Penn-Kekana, Joy Elizabeth Lawn

**Affiliations:** 1 London School of Hygiene & Tropical Medicine, London, United Kingdom; 2 Medical Research Council Unit The Gambia at London School of Hygiene & Tropical Medicine, Fajara, The Gambia

**Keywords:** neonate, newborn, preterm, low birth weight, kangaroo mother care, skin-to-skin, family, grandmother, female relative, gender, qualitative, in-depth interviews, The Gambia, West Africa

## Abstract

Family support is essential for kangaroo mother care (KMC), but there is limited research regarding perceptions of female relatives, and none published from West African contexts. In-depth interviews were conducted from July to August 2017 with a purposive sample of 11 female relatives of preterm neonates admitted to The Gambia’s referral hospital. Data were coded in NVivo 11, and thematic analysis was conducted applying an inductive framework. Female relatives were willing to support mothers by providing KMC and assisting with domestic chores and agricultural labor. Three themes were identified: (a) collective family responsibility for newborn care, with elder relatives being key decision makers, (b) balance between maintaining traditional practices and acceptance of KMC as a medical innovation, and (c) gendered expectations of women’s responsibilities postnatally. Female relatives are influential stakeholders and could play important roles in KMC programs, encourage community ownership, and contribute to improved outcomes for vulnerable newborns.

## Background

Every year, nearly 15 million newborns are born preterm (<37 weeks of gestation; Blencowe et al., 2012) closely linked with 20 million born with a low birth weight (LBW; birth weight <2,500 g; Blencowe et al., 2019). The majority of these vulnerable newborns are born in South-East Asia and sub-Saharan Africa (Ashorn et al., 2020). The smallest and most preterm newborns have the highest risk of illness and death during the first month after birth (Lawn et al., 2014) with complications of prematurity now being the most common direct cause of death in childhood (United Nations Inter-Agency Group for Child Mortality Estimation [UNIGME], 2020). Despite the high risk of preterm or LBW birth, in Africa there is a critical gap in hospital care of small and sick newborns which must be addressed if newborn and child survival targets, now included in the Sustainable Development Goals, are to be met by 2030 (World Health Organization [WHO], 2020).

Kangaroo mother care (KMC) is a package of care provided by caregivers, mostly the mother, in which small newborns receive prolonged skin-to-skin contact (Vesel et al., 2015) in the “kangaroo position.” KMC provides warmth, promotes exclusive breast milk feeding and weight gain, and reduces the risk of infections, often resulting in shorter hospital stay (Conde-Agudelo & Diaz-Rossello, 2016). KMC is recommended as standard care for all stable newborns (birth weight ≤2,000 g; WHO, 2015b) and has the potential to save an estimated 450,000 newborn lives per year (Bhutta et al., 2014) given a 40% mortality reduction with KMC compared to incubator care (Conde-Agudelo & Diaz-Rossello, 2016). Being in the kangaroo position also reduces stress for both KMC provider and newborn, helps manage neonatal pain (Gibbins et al., 2015) and promotes bonding and positive parental mental health (Mörelius et al., 2015). Long-term benefits of KMC have also been reported with decreased hyperactivity and less school absenteeism (Charpak et al., 2017), indicating positive lifelong effects for small newborns after KMC.

Despite first descriptions in Colombia four decades ago (Ray-Sanabria & Martinez-Gomez, 1986) and strong evidence of benefit (Conde-Agudelo & Diaz-Rossello, 2016), the global coverage of KMC is generally low (Vesel et al., 2015). Being in the kangaroo position for 20 hours per day (continuous KMC) is recommended for maximum mortality effect (Conde-Agudelo & Diaz-Rossello, 2016). However, continuous KMC is a major commitment for women and more challenging if they are ill, recovering from cesarean sections or had multiple births. Hence, family support to enable provision of continuous KMC is key (Mazumder et al., 2018). Yet, KMC adoption in resource-limited settings, including Africa, is impeded by lack of family support to enable (a) provision of KMC, especially continuously and (b) undertaking other domestic or family responsibilities (Seidman et al., 2015). Post-discharge continuation of KMC with regular hospital follow-up is an essential part of KMC and also requires buy-in and considerable support from the family and community. Understanding family perceptions toward KMC is also important for community ownership, a key health system bottleneck for KMC implementation (Vesel et al., 2015).

There is limited published data regarding perceptions of family members toward KMC (Kambarami et al., 2002; Seidman et al., 2015; Smith et al., 2017), particularly for grandmothers and other female relatives who play a central advisory role to mothers on newborn care practices (MacDonald et al., 2020; O’Neill et al., 2017). We found no published research on this topic specifically from West African contexts.

The purpose of this study was to understand the perceptions of female relatives toward KMC in a resource-limited setting in which KMC had not yet been implemented. A specific objective was to explore the feasibility and acceptability of female relatives acting as substitute or surrogate KMC providers for small, vulnerable newborns.

**Figure 1.**
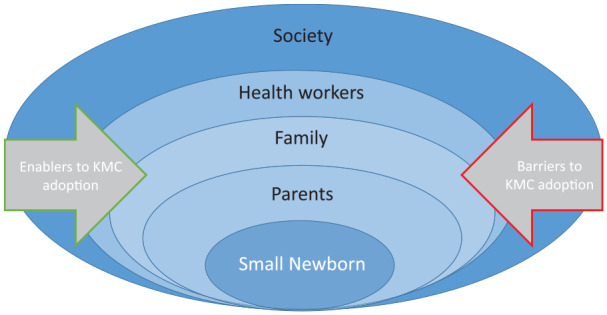
Conceptual framework for implementation of kangaroo mother care (KMC), considering the layers of key stakeholders.

We were guided by a conceptual framework which proposes that KMC implementation can be considered at three levels: (a) mothers, fathers, and families; (b) health-care workers; and (c) facilities (Chan et al., 2016). We further developed and refined this framework to examine the layers of interpersonal and intrasocietal influences on the key stakeholders involved in KMC implementation (Figure 1). Using this conceptual framework, we considered the perspectives of female relatives within the context of the other layers of the model, especially the mothers, and sought to identify barriers and enablers to adoption of KMC from their perspective and within their own systems of adaption, cultural norms, and means of access.

## Method

Based on the concept that perceptions of newborns and their care are influenced by social phenomena, we aimed to construct accounts of participants’ experience, which were collected using in-depth interviews, observations, and reflexive field notes. The study formed part of the formative phase for a randomized controlled trial investigating KMC before stabilization in neonates weighing <2,000 g (Brotherton et al., 2020).

### Political Economy and Household Structure in The Gambia

The Gambia is the smallest country on mainland Africa, with the fourth highest population density and population of ~2.08 million in 2017 (The Gambia Bureau of Statistics, 2019). The predominant religion is Islam (90%), and the Mandinka are the largest ethnic group (35%) followed by Fula (25%), Wolof (15%), and Jola (10%; The Gambia Bureau of Statistics and ICF International, 2014). Polygamy is commonplace, and the most common household structure for all ethnic groups is patrilineal, multigenerational, and extended family groups residing within a compound of varying size, depending on the domestic cycle (Kea, 2013). Compounds typically include a husband, his wives, his married sons, and their wives and children, any unmarried children, widows or divorced sisters, and other extended family (Kea, 2013; Sear et al., 2002).

### Neonatal Morbidity and Mortality in The Gambia

The neonatal mortality rate in The Gambia reduced from 49/1,000 live births in 1990 to 26/1,000 live births in 2018 (UNIGME, 2019). Approximately 17% of Gambian neonates were born LBW in 2015 (UNIGME, 2019) and 12% born preterm (Chawanpaiboon et al., 2019), with complications of prematurity the second most common cause of neonatal death at the neonatal referral hospital (Okomo et al., 2015).

### Hospital Setting

The study took place at the Edward Francis Small Teaching Hospital (EFSTH) neonatal unit, the national neonatal referral unit and the only teaching hospital in The Gambia. Newborns were admitted to the study site from the EFSTH maternity unit (approximately 7,000 births per year) or from other hospitals or home with two thirds of admissions born outside EFSTH (Okomo et al., 2015). At the time of this study in 2017, KMC was not practiced at EFSTH nor widely in The Gambia (Ekholuenetale et al., 2020), although mothers were advised to provide skin-to-skin contact for 30 minutes immediately after feeding for clinically stable, small newborns. Small or sick newborns were cared for under radiant heaters or incubators, often with multiple occupancy, and with few nurses.

### Sampling

Eligible participants were adult (>18 years) female relatives of newborns weighing ≤2,000 g who were admitted to the study site between April and July 2017. We used purposive sampling to identify participants by approaching mothers of currently hospitalized neonates or those discharged within the preceding month. They were contacted by the interviewers in person or by phone, and invitations were extended to their female relatives. Women who were willing to participate contacted the interviewers to arrange a convenient time, and transport expenses were provided. Because different generations and family lines may have different perspectives, we aimed to include maternal and paternal relatives from a range of generations. All participants interviewed were from different families and represented a different neonate. Sample size was based on the availability of participants within the study period.

### Data Collection

Semi-structured interviews were conducted over a 5-week period from July to August 2017 by the interviewers: a non-Gambian female midwife researcher and a multilingual Gambian female field worker. The Gambian interviewer enhanced the credibility of the interviewing team and was able to elucidate and interpret participants’ comments within the cultural context in which they were intended (Guba & Lincoln, 2005). Neither interviewer was involved in the clinical care of the participants or their newborn relatives.

A semi-structured interview guide was used with open-ended questions concerning knowledge and perceptions of newborns, care of small newborns, and KMC (Supplementary File I). Written informed consent, including for audio-recording, was obtained in the participants’ preferred language, with impartial witnesses present for illiterate participants. Informed consent documents were in English, with verbal translation to local languages during the consent process, as per standard local consenting practice in view of the most common local languages having no formal written standard in routine use. Interviews were then conducted in Wolof or Mandinka, as preferred by the participant, in a private, nonclinical room at the hospital. A pictorial information sheet was used to assist the discussion. The interviews lasted between 30 and 40 minutes (average 37 minutes) and were recorded on an ICDPX 440 Sony digital recorder. The interviews were conducted by the same interviewers with the Gambian interviewer leading the interview and the non-Gambian interviewer present for observation of the interview process and reflexivity. The interviewers worked closely together to ensure understanding of the interview guide, and both were experienced in conducting interviews, including on similar topics. The interviewers were aware that as interviews were conducted in the hospital, participants possibly associated the study with the hospital and despite assurances of confidentiality and independence, this may have led to participants sharing what they thought the interviewers wanted to hear. To try and address this, we attempted to build rapport using a warmup session, and the semi-structured interview style allowed participants to lead portions of the interview. As only one interviewer conducted interviews, we were confident that internal validity of the questions was maintained between sessions.

A pilot of two interviews was used to refine the interview guide and to ensure that the Gambian interviewer was familiar with the guide and able to readily translate into the spoken language. After each interview, the interviewers debriefed, which helped maintain reflexivity, improved interview technique, and challenged established assumptions during the analysis and writing. A field diary was kept to document the context and reflections from the interviews, informal conversations with hospital staff and insights into potential findings. Interviews were translated and transcribed into written English text by the same interviewers to ensure consistency and dependability (Tuckett, 2005). Three randomly selected transcripts underwent validation by an independent research nurse fluent in the local languages and English to monitor for accuracy of translation, and no major discrepancies were identified. The use of these research strategies contributed to the rigor of the data collection, especially the reliability and internal validity of data collected (Guba & Lincoln, 2005).

All participants’ data were pseudonymized from the time of enrollment with unique study identification codes for confidentiality. All recordings were deleted from the recorder after transcription. Recordings and transcripts were securely stored on an access-restricted, central server at London School of Hygiene & Tropical Medicine (LSHTM). Ethical approval was obtained from the ethics committees at LSHTM (Ref. 12398) and The Gambia Government/Medical Research Council Joint Ethics Committee (Ref. 1535).

### Analysis

Thematic analysis was conducted using an inductive framework (Braun & Clarke, 2006), allowing codes and themes to develop directly from the data. Due to time constraints, the full transcripts were read and coded by one researcher (the non-Gambian midwife interviewer), who then worked in a cell of qualitative researchers to map, reflect, and refine codes and interpretations of themes. This process was used to help strengthen the reliability of the coding (Guba & Lincoln, 2005). Transcripts were read twice with line-by-line coding on the third reading using NVivo 11 qualitative data analysis software (QSR International Pty Ltd.). The fourth reading focused on merging and reorganizing codes and examining unexpected findings and discrepancies. Codes were then collated into themes, which were refined through iterative analysis and thematic mapping. Themes evolved both directly from the data on a semantic level from explicit meanings and a latent level from interpretation of underlying patterns and ideas (Braun & Clarke, 2006). Quotes were selected to reflect the refined themes. This article was prepared in consultation with Standards for Reporting Qualitative Research (O’Brien et al., 2014).

## Results

### Participant Characteristics

In total, 11 women, representing three generations of maternal and paternal family lines, were interviewed. The relatives consisted of seven grandmothers or great-grandmothers (both paternal and maternal) and four aunts (all maternal). Two women had no children of their own, and the other nine participants had a mean parity of 6.6 (*SD* = 2.1). The predominant ethnic representation was Mandinka (7/11, 64%), and all participants were practicing Muslims. All participants, except one, were resident in a rural region at time of interview. Only two women had attended secondary school, with the remainder receiving only primary level (3/11, 27%) or no formal education (6/11, 55%). All women worked in manual or informal employment, such as market traders, subsistence farmers, or housewives. Most (10 of 11) participants were related to a current in-patient and one to a recently discharged newborn.

### Themes and Perceptions

Three interlinked themes were identified, which gave insight into both contemporary attitudes to newborn care in The Gambia and the acceptability and feasibility of Gambian female relatives supporting KMC. Barriers and enablers for KMC provision and support by female relatives were identified for each theme (Table 1).

**Table 1.** Barriers and Enablers for Adoption of KMC, as Perceived by Female Relatives of Newborns in The Gambia.

Theme	Barriers to KMC Adoption	Enablers of KMC Adoption
1. Collective family responsibility for newborn care practices, including KMC	Lack of buy-in and acceptance by female elders. Fathers too busy to be involved and may not understand the importance of KMC.	Newborn care is a shared responsibility for all female relatives. Elder female relatives are key decision makers for newborn care. Flexibility within the extended family can support the mother (“step-in” roles). Helping mothers with KMC is a reinforcement for positive family relationships.
2. Evolving traditions and the role of medical innovation in acceptance of KMC	KMC is viewed as being different to traditional newborn care practices. KMC (carrying baby on front) is viewed as a Western practice. Uninformed & negative community perceptions of KMC may prevent adoption.	Small newborns are exempt from traditional newborn practices. The KMC wrapper protects small newborns from exposure to evil spirits or “foul wind.” KMC is viewed as a prescribed treatment not a traditional practice. Respect for medical authority
3. Societal expectations of women’s roles and responsibilities in the postnatal period	The physical requirements of KMC will interfere with the ability to perform domestic duties. Women will be unable to do farming work at the same time as KMC. KMC is an additional obligation for the female relative, who has her own domestic & labor responsibilities.	KMC is part of women’s responsibility to protect the newborn from harm. Female relatives have a responsibility to support mothers, which extends to KMC and domestic chores. Intra-household task sharing allows for shifting of domestic responsibilities between women. Female elders can use their authority to task-shift within the compound or family.

*Note.* KMC = kangaroo mother care.

#### Theme 1: Collective family responsibility for newborn care practices, including KMC

Participants identified themselves as part of a larger collaborative unit with a unified, shared responsibility for newborn care. This collective identity was reflected in the cooperative processes of cooking, farming, and health decisions occurring within a compound in which the extended family reside. Participants identified their family as a single unit, with a shared responsibility for the maintenance of the family’s well-being and prosperity:

Myself, my husband and my co-wife and my co-wife’s elder son. We all join hands to care for our children. We are all united. (Maternal grandmother, market trader, Parity 8)

Although all members of the family were considered essential parts of the family unit, participants outlined gendered divisions of responsibility and a general deference to the authority of elder family members in most matters, including knowledge and inheritance of skills. With regard to newborn and maternal health decision-making, this authority was the domain of female relatives, with a hierarchy based on increasing age and elder females holding substantial influence for the care of mothers and their babies. The paternal grandmother of the baby was regarded as the most respected authority for advice on postnatal care and participants deferred to her as the senior authority. This hierarchy has a practical flexibility, and in the absence of the paternal grandmother, other female relatives assume the role, particularly the maternal grandmother followed by co-wives (other wives of the grandfather) or maternal aunts:

That is the role of her mother-in-law, or her own mother or father-in-law and sisters-in-law. They should help the new mother, but if the mother-in-law is less busy, she should be the right person to help. (Paternal grandmother, farmer, Parity 8)

Mothers-in-law should come to support their daughters-in-law, but for our case my granddaughter’s mother-in-law is not there. That is why I am here to support her. (Great grandmother, farmer, Parity 7)

Senior family members took pride and ownership in their role as care providers and were eager to engage in any form of newborn care:

If I wasn’t here [in hospital], things would not work well. I was the one giving care to the baby. (Maternal grandmother, farmer, Parity 10)

Elder female relatives had learned newborn care practices from their own elders, and it was perceived as their responsibility to maintain this system of knowledge inheritance. The authority was explained by seniority of experience accumulated in caring for their own children and other mothers, and they described mothers and newborns as being “under the care” or “the responsibility of” the mother-in-law:

That is the role of we, the eldest, that is our responsibility. We need to advise them, because they don’t have experience on caring for a baby. (Maternal grandmother, farmer, Parity 5)

The physical structure of the compound was described as a hub of family life and a geographical determination of unity, authority, and responsibility. When a new mother returned to the compound of her in-law relatives, she was placed under the umbrella of their advice and protection, and her own mother assumed that role if she returned to her parents’ compound. Nearly all participants expressed the importance of the wider family and community acceptance of KMC, indicating that until others around them in the community were aware of the benefits, KMC would not be encouraged.

Female relatives on both maternal and paternal sides expressed satisfaction and a sense of empowerment after learning about KMC during the interview. They viewed their own and other female relatives’ participation in KMC as a fulfillment of their obligations to their relatives and a validation of their position as an authority on newborns:

Grandmothers will feel happy about it, the same thing applies to great grandmothers, they will say that they have taken care of their children and their children’s children. I’m sure they will be happy about it. (Maternal grandmother, farmer, Parity 10)

Overwhelmingly, the participants agreed in principle to provide KMC themselves in the absence of the mother, whether they viewed the task to be difficult or easy, because of their identified sense of duty to family health:

I will say yes and I will be willing to do it, because I know it is good for the baby. (Maternal grandmother, farmer, Parity 5)

I will say yes, because the baby is my grandchild, I will help my daughter to give kangaroo care to the baby. (Maternal grandmother, farmer, Parity 10)

In addition, their potential contributions to KMC were described as a salve or reinforcement to intra-household relations:

Our relationship would be good, because if you help your relative to do it [KMC] she will know that you like her and her baby. (Maternal aunt, cook, Parity 6)

The provision of KMC was predominantly seen as being within the domain of the mother and the female relatives. Participants’ had mixed views towards the involvement of fathers or male relatives in KMC. Men were deemed as being too busy to participate, or that KMC was something a man would have to do in private, away from the gaze of neighbors.

#### Theme 2: Evolving traditions and the role of medical innovation in acceptance of KMC

An important theme that emerged was the balance between maintaining tradition and embracing new practices, such as KMC, which was viewed as a medical innovation. Female relatives expressed a sense of value and honor in maintaining traditional methods of caring for mothers and newborns, especially traditions involving the extended family structures and seasonal agricultural lifestyles:

In our culture, I mean we the Mandinka, if a woman gives birth if there is an elderly person in the compound, [the elderly person] will be responsible to bathe the baby for one week after birth, after the naming ceremony the in-law or mother to the new mother can take over from the elderly person. (Paternal grandmother, farmer, Parity 7)

However, participants also discussed that some traditions were changing or were no longer valuable, with identification of practices that had changed for the better, such as the use of disposable diapers, provision of antenatal care, and avoidance of traditional medicine, all of which had improved the lives of mothers:

Yes, there is a difference they make good use of the herbs, and God help them to recovery, but now we have health centre everywhere where people go for treatment, during the time of my mother’s they didn’t go for antenatal check-ups. (Paternal grandmother, farmer, Parity 7)

Although most agreed that newborn care practices had changed since the time of their grandmothers’ pregnancies, participants expressed a hesitancy to embrace practices they sensed might affect traditions that were valued, and they articulated concern regarding the perception of their community:

Maybe sometimes they [community/neighbors] will feel like carrying the baby in this position is not good . . . but I think they should know the importance of kangaroo care. (Maternal aunt, cook, Parity 6)

##### Traditions regarding care of small newborns

Small newborns, described as “babies not yet due” regardless of gestational age, were seen as particularly vulnerable to illness, both those transmitted through biomedical and supernatural means. Small newborns were also referred to as “water babies” and were considered not to be fully formed humans, leading to high risk of illness and death from supernatural means such as wind (bad air) or *Jinne* (evil spirits). It was believed that using physical barriers, such as fabric to wrap or swaddle a baby, protects small newborns from such supernatural forces:

I heard it from the elders, that they [small newborns] should not be exposed to the public, if exposed they become sick easily and pass away. (Maternal aunt, market trader, Parity 0)

A baby should be wrapped to protect her from evil eyes, they [small newborns] are called water babies, the moment the eyes are set on them they pass away. (Maternal aunt, market trader, Parity 0)

Despite the prevalence of preterm and LBW babies born in The Gambia, none of the participants reported caring for or seeing a small newborn prior to the current admission, and many noted that a vulnerable, small newborn should be hidden from the community.

Care of the small newborn was viewed differently from usual newborn care, and therefore was flexible to the many requirements of traditional newborn care practices:

I think babies not yet due should be wiped with a clean cloth, and they should be wrapped with heavy wrappers [fabric]. When it comes to feeding some of them cannot suck breast, I think they should be spoon-fed. (Maternal grandmother, farmer, Parity 5)

Wrapping the newborn in numerous pieces of fabric was seen as routine for all newborns but emphasized for small newborns as a means to defend against air and subsequent illness:

Babies not yet due should not be bathed, their body should be wiped, too much water is not good for them, they need to be wrapped [in fabric] and should not be exposed to the air . . . Mothers need to be very careful of their babies not yet due. (Great grandmother, farmer, Parity 7)

There was knowledge and understanding of the higher risks of mortality associated with being born small, with some participants associating the likelihood of survival with religious beliefs:

At the moment I am praising God at all times, because God gave it [the baby] to us. That’s what I have in my mind, but I really thank God, I am praying . . . for them to survive. (Maternal grandmother, farmer, Parity 5)

##### Acceptability of KMC

Participants frequently commented that KMC was a Western practice, in reference to front baby carriers popular in Western countries. This contrasts with the traditional Gambian method of swaddling babies in fabric and holding them in arms for the first month after birth, after which they are carried on the caregiver’s back. Female relatives voiced apprehension that they would be viewed as abandoning tradition:

Yes, I see the white people carrying their baby this way. I first saw it with the white people . . . Well, if I don’t know it, I will think they are copying the white people. Because, we know of the white people carrying their baby in front, we carry our babies on our back. (Great grandmother, farmer, Parity 7)

You know I have seen it, but if the [other mothers] didn’t see the image [KMC information sheet] they might not know that the baby is born before its time, they may think the mother is copying the western culture. (Paternal grandmother, housewife, Parity 6)

Carrying newborns on the back was viewed both as a convenience and a way by which to protect the child from harm, as the mother is physically in front of the baby. Concerns were expressed about the vulnerability of the newborns position between the mother’s breasts, with a perception that the newborn was more vulnerable to harm in the KMC position:

If you carry the baby in the kangaroo position, you need to be careful not to fall down. (Paternal grandmother, farmer, Parity 8)

It is safer when you carry the baby on your back and tie the wrapper [fabric] properly, nothing will happen to the baby. (Paternal grandmother, farmer, Parity 7)

Despite the differences with traditional practices and reservations about safety, there was acceptance of KMC and a willingness to provide KMC themselves because it was seen as a care practice specifically for small newborns, rather than an attempt to alter traditional practices. The authority of health workers in recommending KMC was also identified as promoting KMC acceptance:

They will accept it if it is the advice given by doctors. No, that [KMC] will not be a problem at home, it has nothing to do with traditions, if you are asked why, you will let them know it is the advice given to you by doctors for babies not yet due. (Great grandmother, farmer, Parity 7)

Overwhelmingly, innovative changes to practices such as KMC were seen as helpful and a positive change both for the participants and their families:

I think they should like it [KMC]. I don’t think it should have to be with tradition, people don’t care for tradition that much now. The health of the baby is the most important thing. (Paternal grandmother, farmer, Parity 8)

In addition, participants identified similarities between KMC and traditional newborn care practices, especially the importance of keeping babies warm and protecting them from exposure to air. Many participants embraced the idea that KMC could include an outer cloth around the back of the baby and accepted this as a protective practice, similar to how newborns are traditionally wrapped in fabric.

#### Theme 3: Societal expectations of women’s roles and responsibilities in the postnatal period

The acceptability and feasibility of KMC was rationalized through the lens of how it would influence or disrupt the expected roles and responsibilities of women (mothers and female relatives) during the postnatal period. It was acknowledged that women’s responsibilities change with advancing age, with core responsibilities including obligations to family, God, housework, and, for some, farming.

##### Responsibility to protect the newborn

Mothers are exempt from many physical duties during the first 40 days following delivery but are expected to be the primary carer for their newborn, with the support of elder female relatives. The mother’s foremost responsibility was the protection of her child, expressed through shielding the newborn from causes of illness, both biomedical and supernatural. Acts such as wrapping, keeping the baby under a mosquito net, and carrying the baby on her back were all described as physical barriers meant to protect the newborn. The physicality of KMC was identified as a potential challenge, but KMC was viewed as another method of protecting the newborn and fulfilling their responsibilities:

It [KMC] is good for the baby . . . because when you carry your baby in front, you will be able to notice her at all times. (Maternal aunt, cook, Parity 6)

Maintaining good hygiene was also highly valued as a ritual and responsibility to ensure health for the newborn:

The mother needs to be clean always so that the baby will be healthy. If the baby is not healthy, her mother will not be free. (Paternal grandmother, farmer, Parity 8)

Person doing kangaroo care should put on clean cloths [fabric used to secure baby in KMC position], and pay attention to the baby, the cloths should be clean always. (Paternal grandmother, housewife, Parity 6)

##### Domestic responsibilities and KMC provision

Domestic duties were the central responsibility for women of reproductive age, and this was observed as a barrier to providing KMC after hospital discharge:

She should always be careful when she is doing household work, and she should know the type of household work she can do during kangaroo care. (Maternal grandmother, farmer, Parity 5)

There was an understanding that some domestic duties were still possible in combination with KMC and that appropriate education for the mother and relatives would be helpful:

During kangaroo care you will be able to walk around the compound, this is just like carrying the baby on your back, although you cannot bend down, but you will be able to do certain work . . . during kangaroo care you cannot cook with the baby, or bend down with the baby, you cannot pound [grain]. (Great grandmother, farmer, Parity 7)

KMC was perceived as an additional obligation for the female relative who supports the mother. However, participants were willing to modify some of their own responsibilities to accommodate the needs of the mother and newborn.

##### Farming obligations and support of female relatives

Many participants had strong obligations to farming, and any theoretical contributions to providing KMC were linked to the agricultural calendar. Participants described some flexibility within the household dynamics which would allow for a negotiation of labor contributions. Female elders suggested that they could use their authority to modify the expected labor and domestic contributions of KMC practicing mothers and task-shift (ask other family members to do the mother’s domestic responsibilities) with other family or household members. There was a willingness and a flexibility from female relatives to provide KMC when they weren’t farming:

We can give kangaroo care before going to the farm in the morning, or we can give kangaroo in the afternoon, when we come back. (Maternal grandmother, farmer, Parity 10)

KMC was viewed as being consistent with the expected societal roles and responsibilities during the postnatal period and was viewed as feasible within both hospital and community settings.

## Discussion

This in-depth, qualitative study of female relatives’ perceptions of newborn care and KMC is consistent with recent calls from the WHO to conduct formative research from a family-systems perspective to improve maternal and newborn implementation programs (WHO, 2015a) and address evidence gaps relevant to implementation of high impact care (Smith et al., 2017).

We identified overarching themes of (a) collective family responsibility, (b) evolving traditions and the role of medical innovation, and (c) societal expectations of women during the postnatal period, which provide important insights into the barriers and enablers for adoption and support of KMC by female relatives (Table 1). These themes are consistent with many of those previously identified as being important for KMC adoption in previous systematic reviews of the topic, especially KMC provider buy-in and bonding, social support, and cultural context (Chan et al., 2016; Smith et al., 2017).

Historically, child health interventions have focused on the mother–child relationship without consideration of the social structures and cultural systems that influence health beliefs and behaviors (Aubel, 2014). By considering the views of family members within the context of the other layers of KMC implementation (Figure 1), we appreciate how kinship bonds among women are central to KMC practice and acceptance.

We identified an overwhelming acceptance and willingness for female relatives to support mothers, both by providing KMC themselves and assisting the mother with domestic and labor responsibilities. Shared familial responsibility, intergenerational relationships, and deference to elder female authority were key enablers for newborn care and KMC provision. The finding that women were motivated to help their relatives in an effort to reinforce intra-household relationships is consistent with the theory that kin relationships can develop through everyday experiences within the domestic sphere (Carsten, 2000).

Our finding that elder female relatives are influential for newborn care is well described in many African contexts (Aubel et al., 2004; Iganus et al., 2015; MacDonald et al., 2020) and highlights these women as key actors for the uptake and continuation of public health interventions such as KMC (Gupta et al., 2015; Iganus et al., 2015; Mazumder et al., 2018). Programs that utilize both the decision-making influence and caretaking role of elder family members have the potential to change behavior more effectively (Iganus et al., 2015). This is in-keeping with The Every Newborn Action Plan, which advocates to incorporate influential family members, such as grandmothers, to strengthen support networks for newborn care (WHO, United Nations Children’s Fund [UNICEF], 2014).

The acceptance of KMC by female relatives is balanced between respecting traditional beliefs and viewing KMC as a prescribed treatment or medical innovation for small newborns. This is linked to an observed respect for the authority of health workers. When considered through the lens of our conceptual framework (Figure 1), the wider social hierarchy and context-specific relationships between health worker and family member are key to understand and account for so that appropriate sensitization and implementation methods can be used. Ensuring adequate health worker education and knowledge of KMC is important in the West African context so as to support the female relatives’ buy-in and acceptance.

Our findings support those from other African studies that carrying a newborn in front is viewed as representative of Western customs is contrary to traditional African newborn practices and is a potential barrier to KMC practice (Chan et al., 2016; Smith et al., 2017). Those aspects of KMC which are incongruous with local practice, such as carrying the newborn on the front, should be sensitive to their potential cultural implications and efforts should be made to include the biomedical explanations and benefits in community and hospital-based KMC sensitization activities.

Our findings around the need to protect newborns from the bad air or evil spirits reflect a common traditional belief in The Gambia and elsewhere that “foul wind” may be harmful to newborns and it is necessary to cover and protect the baby from *Jinne* or illness (Baum et al., 2012; O’Neill et al., 2017). Protecting small newborns by securing the baby in KMC position with a wrapper is consistent with these traditional beliefs and is a potential enabler to promote KMC practice. Kumar and colleagues (2008) used a similar technique in India for the improvement of newborn survival by merging key messages with existing beliefs and practices to facilitate behavior change.

Women undertaking caring responsibilities for family members have previously been described as “women in the middle of competing role demands, competing generations, and competing emotions” (Roe et al., 1994). This is reflected in our observation that women’s postnatal responsibilities are centered on the care and support of the newborn and mother, but also encompass domestic and agricultural obligations within the intergenerational household sphere. Navigating these competing roles and understanding how KMC practice impacts women’s postnatal responsibilities is essential for KMC programs and to promote continuation of KMC after hospital discharge. We identified that KMC is consistent with women’s expected responsibilities but the impact for those with agricultural livelihoods should be considered further and opportunities for encouraging task-shifting within the family or household explored. Although impact on domestic chores is a well described barrier to KMC (Seidman et al., 2015; Smith et al., 2017), the negative effects on KMC practice have not been established in previous research (Nguah et al., 2011) and support from female relatives is an important mitigating factor.

The study has several strengths. It provides a detailed insight into perceptions of a previously underrepresented population around important newborn care practices. Despite the small sample size, thematic saturation was reached, and the findings provide a rich and detailed understanding of women’s perceptions of newborn care in contemporary West Africa. The findings are transferable to other contexts with similar polygamous, patrilineal, and multigenerational household structures and gendered societal expectations. The data are dependable due to research and operational techniques and adheres to established trustworthiness criteria (Tuckett, 2005).

However, because KMC was not routinely practiced at time of the study, the findings reflect female relative’s perceptions rather than their direct experience or behaviors. Thus, it provides insight for a setting which is KMC-naïve. Given the small sample size, these participants represent only a subsection of relatives who were willing to participate, and hence generalizability may be limited to female relatives already engaged with the hospital and invested in the care of their relative’s newborn. Thus, the findings may not be representative of other female relatives in the community or those who choose not to accompany their relatives to hospital. Social desirability bias is also a risk due to the association of the interviewers with a locally well-regarded research institution (Medical Research Council Unit The Gambia at the London School of Hygiene & Tropical Medicine [MRCG]) and with the hospital. The interviews were coded by a single researcher which may have led to the analysis being shaped by her own perspectives and understanding.

Maternal perceptions of newborn care and KMC are well-documented, but further research is required to understand fathers’ perceptions, as their influence and support for mothers and female relatives is also key to the success of antenatal and postnatal health programs (Audet et al., 2016). For a holistic understanding of barriers and enablers to KMC in West Africa, the voices of other key stakeholders such as health workers, policy makers, community and religious leaders are also needed to encourage participation and buy-in with the aim of supporting further KMC roll-out. Further understanding of the interpersonal and power dynamics between health workers and families would provide valuable insights for behavioral sciences and implementation science approaches to promoting KMC uptake.

## Conclusion

We identified that in the Gambian context, female relatives of hospitalized small newborns accept KMC and are willing to both provide KMC themselves and support the mother with her postnatal responsibilities. Our findings add to the evidence that mothers in Africa are not autonomous decision makers, and female relatives are important stakeholders in newborn care decision-making and practices. Recognition of the importance of female relatives may create more holistic, family-centered approaches to implementation of newborn public health interventions. These women’s voices have the power to identify and address barriers and enablers for more widespread adoption of KMC as a life-saving intervention for small, vulnerable newborns.

## Supplemental Material

sj-pdf-1-qhr-10.1177_1049732320976365 – Supplemental material for “We All Join Hands”: Perceptions of the Kangaroo Method Among Female Relatives of Newborns in The Gambia Supplemental material, sj-pdf-1-qhr-10.1177_1049732320976365 for “We All Join Hands”: Perceptions of the Kangaroo Method Among Female Relatives of Newborns in The Gambia by Helen Brotherton, Maura Daly, Penda Johm, Bintou Jarju, Joanna Schellenberg, Loveday Penn-Kekana and Joy Elizabeth Lawn in Qualitative Health Research
