# Supplementary material for: “We All Join Hands”: Perceptions of the Kangaroo Method Among Female Relatives of Newborns in The Gambia
Source: Qual Health Res. 2020 Dec 8;31(4):665–76. doi: 10.1177/1049732320976365 (PMC7882999; doi:10.1177/1049732320976365)
Supplement: sj-pdf-1-qhr-10.1177_1049732320976365 – Supplemental material for “We All Join Hands”: Perceptions of the Kangaroo Method Among Female Relatives of Newborns in The Gambia [file sj-pdf-1-qhr-10.1177_1049732320976365.pdf]

Supplementary file I: Interview Guide

Female Family Kangaroo
In-Depth Interview Guide

Outline :

I. *Introduction*
II. *Warm-up questions*
III. *Newborn Knowledge*
IV. *Low Birthweight and preterm knowledge*
V. *Kangaroo care knowledge*

I. ***Introduction:***

Thank you for agreeing to take part in this interview. My name is .... I am now going to ask you some questions regarding your experiences with caring for newborns, small babies or babies born too early and kangaroo care. As explained, you are free to stop the interview at any time. This interview will be audio-recorded (tape recorded) and will take approximately 40 minutes to an hour. Again, I just want to emphasize that we just want to know about your experiences and thoughts. There are no right or wrong answers; we just want to hear what you think. Thank you for your time.

II. Warm-up questions / Background

Tell me a little bit about yourself. Where were you born? Do you have any children? [Just build up a rapport]
Background:

- Are you married / divorced / widowed / never married?
- Did you finish high school? Did you complete more education after high school?
- What is your religion?
- Do you work? What is your occupation?
- Who do you live with?
- How many children do you have?
- Who helps you to take care of your children?
- Do you have a family member that has had a small or born too early baby?

III. Newborn knowledge

What are the usual ways to care for a newborn?

- Probes: Talk about their experience with newborns, with their own and others.

What does newborn care mean to you?

- Probes: Explore following aspects of newborn care

- When should a baby be bathed? By whom?
- How do you care for the cord? What should be put on it and by whom?
- How do you keep a baby warm, should the baby be wrapped?
- What are the reasons a baby can become sick?
- Who usually helps a newborn mother care for her baby?
- Who should a mother trust to take care of her children when she is not at home?

What are things you can do to make the baby healthy?

Who usually gives new mothers and fathers advice about caring for a baby? Who gives you advice?

What does a good mother do when caring for a newborn?

Has the way you care for newborns changed over time?
- Probes: Did your grandmother or mother do things differently?

What are the typical ways to care for a new mother?
- Probes: Who usually accompanies and supports her? Who gives her advice?
- When a mother and new baby leave the hospital, which house or compound do they go to?

IV. Experience and perceptions of low birthweight or preterm newborns

Now I want to know about your experience with small babies or babies born too early

Tell me about the baby you are related to that is in the neonatal unit, why is the baby here?

How do you feel about the baby being in the neonatal unit?

How often have you been here since the baby was admitted?

Do you think being here is helpful to the baby and mother?

Who do you think should come to support the mother and baby?

Why do you think babies are born too early or small?

When a woman has a small baby, or baby born too early in the hospital, who usually comes to support her?

Have you ever had a small baby or baby born too early or cared for one?
- **Probes:**
  - If yes: how did you care for the baby?
  - Did you do things differently than for a normal newborn? If so, what?

    - Did you have any trouble caring for the baby? How is it different from normal care? If no; how would you care for the baby?

Would you care differently for twins ?
What are the usual ways to take care of a small bay or babies born too early in your community?

- Probe: Tell me more about this

Are there special ways of caring for small babies? Bathing, feeding?

What is the typical advice a mother of a small baby is given?

How do you know if a baby is too small for normal care?

What are the things mothers or carers have to be most careful about when caring for small babies or babies born too early?

What does the community or neighbours think about small babies or babies born too early? What do other mothers think?

- Probe: Why are they born early? When is too early to survive?

V. Kangaroo Care

Have you heard about or seen kangaroo care?

- Probe: If yes, what is it and where did you first hear about it?
  If no, provide the information sheet and explain concept, check understanding before continuing

Why do you think kangaroo care is done?

Is kangaroo care different from what your mothers or grandmother did with babies born early or small?

What effects do you think kangaroo care has on the baby?

- Probe: is it good or bad?

Why do you think the baby is help in this position?

What effects do you think kangaroo care has on the mother?

If you had a small baby would you choose to do kangaroo care?

- Probe: Why / why not

Do you think kangaroo care is easy or hard?

- Probe: Why and for whom

Are there reasons why it shouldn't / couldn't be done?

How do you think the baby feels, receiving kangaroo care?

How do you think the mother will feel?

Is kangaroo care similar or different to the ways your mother or grandmother would have cared for a small baby?

Do you think it is possible to give kangaroo care for 18 – 20 hours per day?

What are the things mothers or families have to be most careful about when doing kangaroo care?

How do you think the rest of the family feels about kangaroo care?

Is it something the mother will be able to continue at home?

What will be the challenges she will face when doing kangaroo care at home?

How do you feel when you think about kangaroo care?

How would you feel if you were asked to help the mother give kangaroo care to her small baby?

- Probe: Is it different than the normal help you would provide? Easier ? Harder?

Kangaroo care should be given for 18-20 hours a day. Sometimes the mother is tired or recovering from a C-section, or there are twins. If you were asked, how would you feel about providing some of the kangaroo care ?

- Probe: Would you say yes or no?
  If yes, how long would you do it?
  How long do you think the mother would react? The baby?
  Would you do it more than once? If not, why not?
  What circumstances would you do kangaroo care?
  Do you think this is good for the baby ? The mother?

When is the best time for family members to provide kangaroo care?

How often should the family member provide kangaroo care?

Should the father of the baby also provide kangaroo care?

Would your relationship with your family change [if you did kangaroo care] ?

- Probe: If yes, how?

Who besides the mother should or can provide the skin to skin part of kangaroo care?

Who besides the mother or father can provide consent for the baby to receive kangaroo care?

What do you think other mothers will think when they see kangaroo care?

How do you think your community / neighbours will feel about kangaroo care?

Thinking now about all the things we have talked about, is there anything else we haven't discussed that you think is important about kangaroo care?

Thank you so much for taking the time to talk to me. Do you have any questions you would like to ask me?

To Interviewer: Stop recording. Interview ends.
